# Results of a participatory needs assessment demonstrate an opportunity to involve people who use alcohol in drug user activism and harm reduction

**DOI:** 10.1186/s12954-016-0126-x

**Published:** 2016-12-09

**Authors:** Alexis Crabtree, Nicole Latham, Lorna Bird, Jane Buxton

**Affiliations:** 1 British Columbia Centre for Disease Control, Vancouver, Canada; 2 University of British Columbia, Vancouver, Canada; 3 Vancouver Coastal Health, Vancouver, Canada; 4 Vancouver Area Network of Drug Users, Vancouver, Canada

**Keywords:** Illicit drugs, Non-beverage alcohol, Illicit alcohol, Harm reduction, Substance use, Participatory research, Ethnography, Drug users’ organizations

## Abstract

**Background:**

Drug users’ organizations have made progress in recent years in advocating for the health and human rights of people who use illicit drugs but have historically not emphasized the needs of people who drink alcohol.

**Methods:**

This paper reports on a qualitative participatory needs assessment with people who use illicit substances in British Columbia, Canada. We held workshops in 17 communities; these were facilitated by people who use illicit drugs, recorded with ethnographic fieldnotes, and analyzed using critical theory.

**Results:**

Although the workshops were targeted to people who use illicit drugs, people who primarily consume alcohol also attended. An unexpected finding was the potential for drug users’ organizations and other harm reduction programs to involve “illicit drinkers”: people who drink non-beverage alcohol (e.g. mouthwash, rubbing alcohol) and those who drink beverage alcohol in criminalized ways (e.g., homeless drinkers). Potential points of alliance between these groups are common priorities (specifically, improving treatment by health professionals and the police, expanding housing options, and implementing harm reduction services), common values (reducing surveillance and improving accountability of services), and polysubstance use.

**Conclusions:**

Despite these potential points of alliance, there has historically been limited involvement of illicit drinkers in drug users’ activism. Possible barriers to involvement of illicit drinkers in drug users’ organizations include racism (as discourses around alcohol use are highly racialized), horizontal violence, the extreme marginalization of illicit drinkers, and knowledge gaps around harm reduction for alcohol. Understanding the commonalities between people who use drugs and people who use alcohol, as well as the potential barriers to alliance between them, may facilitate the greater involvement of illicit drinkers in drug users’ organizations and harm reduction services.

## Background

Recent years have seen drug users’ organizations, and their allies make substantial progress in advocating for the health and human rights of people who use illicit drugs. Historically, drug users’ organizations (organizations that are led by people who use illicit drugs and that work to improve their lives at individual and systemic levels) have focused their efforts on currently illegal substances, and not prioritized alcohol or the needs of people whose substance of choice is alcohol.

A key theoretical concept in studying the health of people who use illicit substances is structural violence, defined as a cause of suffering that is unnatural and caused by forces external to the individual. The term refers to “historically given (and often economically driven) processes and forces that conspire to constrain individual agency” [1] and lead to an unequal and unjust distribution of suffering. Analysis of instances of structural violence should attend to intersecting oppressions based on gender, ethnicity, class, and other factors but must not lose sight of individual agency in the face of increased risk of suffering within marginalized communities [1, 2].

This paper reports on the results of a participatory needs assessment with drug users^1^ in British Columbia, Canada, in which an unexpected finding was an opportunity to engage marginalized people who drink alcohol in drug user activism and harm reduction. This paper summarizes the results of the needs assessment for drug users; explores the rationale for greater involvement of illicit drinkers in drug user activism based on shared priorities, shared values, and experiences of polysubstance use; and identifies barriers to drug user organizations becoming more inclusive of illicit drinkers. We use the term illicit drinking use to describe the consumption of non-beverage alcohol (alcohol not meant for human consumption, e.g., mouthwash and rubbing alcohol) and the consumption of beverage alcohol in ways that are highly criminalized (e.g., public drinking by people who are homeless).

## Methods

**Fig. 1:**
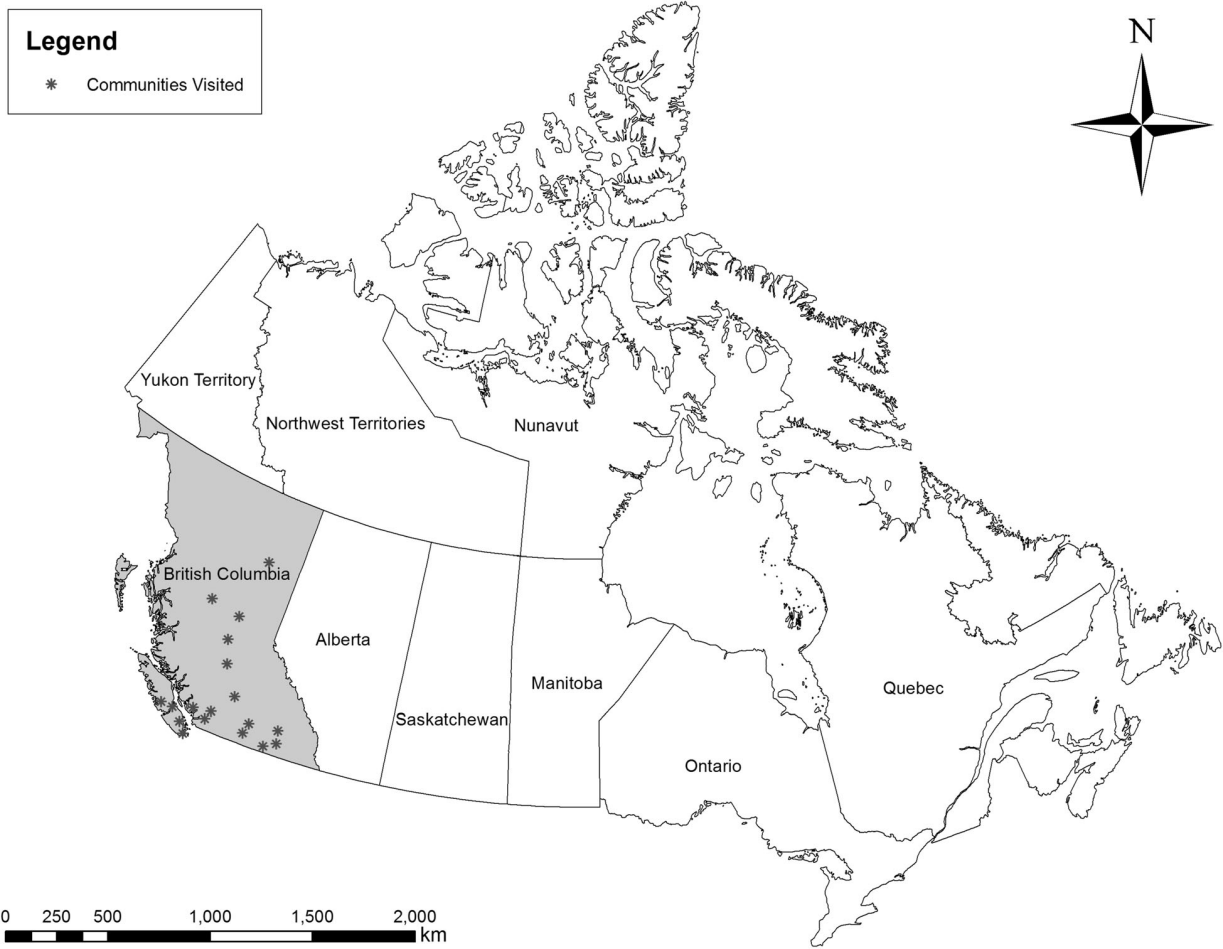
The sites of workshops in the drug users’ needs assessment

This paper describes results obtained as part of a larger qualitative, participatory study to investigate the priorities and values of drug users in the province of British Columbia, Canada. Data collection was focused on communities outside Metro Vancouver, which is the province’s largest metropolitan area, in order to better understand the experiences of people who use drugs in smaller communities and rural areas. For full description of the methodology summarized here, please see [3]. We held a series of 17 workshops in communities around British Columbia (see Fig. 1).

These workshops were advertised to substance users by local harm reduction and health care organizations and were structured around the question “What do drug users need to live healthy lives in their communities?” Although outreach was directed toward drug users, people who primarily consumed alcohol were not precluded from attending provided they identified as current or past users of illicit substances on occasion. Three hundred and two participants attended the workshops, with mean of 18 participants (range 6 to 42) per meeting.

These workshops were facilitated by current or former illicit drug users and a paid staff person from the Vancouver Area Network of Drug Users, a drug users’ organization in Vancouver, British Columbia. Facilitators asked open- and closed-ended questions to elicit discussion about priorities for harm reduction and health promotion among participants, then engaged participants in a discussion of strategies by which progress could be made toward these priorities. Between workshops, the facilitators (including authors NL and LB) and the field researcher (AC) held debriefing sessions to discuss ongoing results and plan specific areas of inquiry for subsequent workshops.

AC took ethnographic fieldnotes at the workshops and following discussions with facilitators between workshops. Based on their experience with this population, the facilitators felt that audio recordings would not be acceptable to many participants and would discourage open discussion. Data analysis took place in two phases. Initial analytic work took place at daily debriefing meetings. While not a formal academic process, these initial reflections impacted data collection and helped develop ideas that were refined in a more traditional process of critical analysis using NVivo 7 [4].

Initial results were presented and feedback solicited at an annual general meeting of the BC-Yukon Association of Drug War Survivors (a regional drug users’ organization), and minor refinement of the results took place in response to feedback received.

## Results

### Summary of drug users’ priorities and values

Workshop discussions led to the development of seven priorities for change that were shared by drug user participants across the province: (1) improving interactions with health professionals, (2) promoting access to a range of housing options, (3) improving treatment by police, (4) ensuring harm reduction best practices are followed everywhere, (5) improving social assistance, (6) supporting drug users’ organizations, and (7) engaging new and existing allies. Participants’ experiences of health care, housing, policing, and social assistance can be seen as examples of the ways in which structural violence is made manifest in drug users’ lives. The remaining three priorities suggested by participants—promotion of user-run organizations, implementation of best practices in harm reduction, and the recruitment of new allies—celebrate the potential of resistance to structural violence. In these areas, participants saw opportunities to build on past victories for drug users and to take further action to improve the health of their communities.

We identified four values underlying drug user participants’ priorities for change: collectivity, activity, freedom from surveillance, and accountability. *Collectivity* involved a desire for connection with others that was irrespective of substance use and entailed responsibility for the welfare of others; participants acknowledged the many barriers they face to achieving this ideal. While *actively self-advocating* was seen as key to achieving the priorities, participants noted that passivity is encouraged by many of the institutions with which they interact (for example, hospitals and police). *Surveillance* was ubiquitous in participants’ lives, and particularly intense in medical settings; they described a paradoxical desire for privacy and “not having to hide” (Community A, hereafter referred to as “A”). Finally, *accountability* was contrasted with surveillance as a system in which drug users would have meaningful influence over the institutions in their lives and would be accountable to them in turn. These values reflect participants’ experiences of dominant ideologies, particularly the emphasis placed under neoliberalism on economic productivity, individualism, and self-monitoring, and the discipline and control of people who do not conform to these expectations [5]. The values also demonstrate the possibilities of responding to these ideologies through resistance and “strategic accommodation” [6].

For a more in-depth discussion of the priorities and values, please see [3].

### Points of alliance between drug users and illicit drinkers

#### Shared priorities

Not all of the priorities that were identified for drug users were shared by the illicit drinkers that attended the workshops. Supporting drug user-run organizations, not surprisingly, was not raised as a priority by these participants, nor was developing alliances or improving social assistance. The other four priorities, however, were repeatedly mentioned by those who identified as illicit drinkers; that is, they expressed a desire to see improved relationships with health professionals, a greater range of housing options, better treatment from police, and implementation of harm reduction services.

Drug user workshop participants described interactions with health professionals that are marred by discrimination and surveillance. Others have similarly found that relationships between health care workers and drug users are characterized by mistrust, resulting in less care-seeking and lower quality therapeutic relationships [7–14]. The illicit drinkers echoed these concerns: they felt that physicians and nurses arrive to patient encounters with pre-formed opinions about the needs and motivations of marginalized clients and therefore are not in a position to provide responsive care. One participant described his experience of being unfairly judged at a hospital:

Like everyone says, it’s hit and miss here. Certain clinics will treat you with respect, [but] the hospital triages you first. I went there for an abscess. I wasn’t using drugs then, only drinking. They made me wait eight hours. They singled me out, they thought I was a drug user and they were all talking. It’s a human rights violation (Community C).

Other illicit drinkers described similar concerns, and, like the drug user participants, placed a high priority on the development of systems to ensure health care could be obtained without judgement or loss of confidentiality.

In addition to their concerns about health services, drug user and illicit drinker participants alike expressed dissatisfaction with the housing options available to them, primarily centered on the need to provide shelter to those who are actively using substances. The following exchange demonstrates the constrained choices faced by drinkers in a town with a single shelter:

Participant 1: A lot of people want to talk about health, but if you’re homeless--

Participant 2: They told me I had to sleep outside because I was drinking.

Participant 3: If you quit the drunkenness, you’ll get a place to sleep overnight!

Participant 1: And if you can’t quit the drunkenness?

Participant 4: You end up sleeping in the bank, in the ATM area (Community O).

Similar to the views expressed by drug users, these drinkers find the restrictions placed on them preventing them from meeting a basic need, a situation which could be ameliorated by providing shelter that allowed for active substance use among that segment of the population unable or unwilling to restrain from it.

In describing the pathways to improved relationships with police, drug user participants mentioned the necessity of decriminalization of drug use and of reformed police conduct in regards to harassment, violence, and protection of drug users. Academic researchers have also identified aspects of policing as detrimental to drug users’ health. In particular, intensive policing of drug markets encourages riskier drug use behaviors and interferes with the use of harm reduction and health services [15–19]. Incarceration is associated with increased morbidity from infectious and non-infectious diseases [20, 21], and negative relationships between police and drug users may contribute to internalized stigma and interfere with recovery [11]. Illicit drinker attendees pointed out that although their substance of choice is legal; they too suffer from the consequences of improper treatment by police. For example, one group of friends explained their frequent interactions with police in their small town:

Participant 1: The cops always harass you, pull up on you, ask you what you’re up to, even if you’re just sitting there.

Participant 2: Even look through your backpacks.

Participant 3: And we don’t have nothing, just a couple empties (Community N).

While these participants expressed their frustration with what they perceived as being inappropriate targets of police scrutiny, others mentioned lack of attention to health needs or even outright violence while in custody as examples of the unacceptable treatment they receive from police. As well, and again similar to the views expressed by drug user participants, illicit drinkers brought up what they saw as a lack of action by the police in protecting them when they are the victims of crime. In one small town, several workshop participants described how they felt the police had been derelict in their duties:

Participant 1: There’s this one gang that goes around town beating up homeless people, and the cops do nothing.

Participant 2: I got shot with a pellet gun by them and the cops didn’t help.

Participant 1: We tell them what kind of cars they drive and still they don’t help us (O).

The description of this incident echoes the sentiment of drug user participants that police are uninterested in protecting the rights and safety of people who use illicit substances. This is in keeping with a view of the criminal justice system as functioning in large part to protect the interests of those in power, a group from which illicit drinkers are clearly excluded.

Finally, drug user workshop participants expressed the belief that harm reduction services do improve their health and well-being, but that these services would be more effective if expanded and always offered in accordance with proven best practices. Illicit drinker participants, too, seemed convinced of the value of harm reduction. Their comments, reflecting the current sparsity of alcohol harm reduction services, emphasized the potential benefits of adapting programs for drug users to meet the needs of those who drink alcohol. For example, after describing the utility of supervised injection spaces, one illicit drinker asked, “What about for people who are alcoholics? They should have a little community or building for people who drink outside so they can be safe inside” (C). Other participants described the need for education on reducing harms from non-beverage alcohol (similar to the educational programs offered to injection drug users) and preventing transmission of infections among people sharing the same bottle (inspired by the distribution of mouthpieces for crack pipes).

#### Shared values

In addition to the shared priorities described above, both drug user and illicit drinker workshop participants strongly expressed their desire to see the existing system of surveillance and judgement from those in authority replaced by one of mutual accountability. Drug user and illicit drinker participants were positioned similarly in their positions relative to institutions of surveillance (e.g. policing, medical, and social assistance), although the difference between illegal and legal substances does necessarily affect the types and sites of surveillance to which they are subjected. Dissatisfaction with ubiquitous surveillance is clear in this young person’s description of an encounter that occurred while she was walking home from classes:

The cops came up to me and said what was I doing outside walking at ten at night? I said, ‘Walking home.’ They said, ‘You been drinking?’ I said, ‘None of your business!’ They just want to take you in. I’m not allowed to walk down the street? (O)

An additional example of surveillance given by participants in multiple communities involved staff and customers at liquor stores. The comments included one participant describing the owner of the community’s only liquor store as being “like a king,” explaining that “he watches everyone outside” (O).

Similar to drug users, illicit drinker participants particularly chafed against the judgement that surveillance of their activities entailed. One woman explained that she drank non-beverage alcohol and said, to an enthusiastic response from the other participants, “There’s folks out there that do drink alcohol, hairspray. You should not judge other people, that’s their right, it’s up to the people, what they drink out there” (C). Another participant described obtaining food at one of the few places available in his community: “When you go to the soup line, if you’re drunk, they kick you out. You just want to eat and they make you eat outside. We’re not dogs!” (N) His last sentence implies that, more than the surveillance for intoxication or the restriction on where he can eat, he reacted negatively to the implied judgment that he is not fit to eat inside.

In contrast to unidirectional surveillance and judgement, some illicit drinkers echoed the comments of drug user participants by voicing their desire for greater say in the institutions that shape their lives. A service organization in one community, for example, feeds people “a bowl of soup and moldy bread” and “you get kicked out for a week for being intoxicated.” Participants wondered, “How do they get funding for that?” (O) This reflects their belief that the organization is not, in their opinion, meeting the mandate for which it receives funds and shows that drug users’ organizations could potentially ally with illicit drinkers to push institutions to be more accountable to the people they serve.

#### Polysubstance use

Until this point, drug users and illicit drinkers have been referred to as if they are separate and distinct categories. The reality of substance use, however, is more complicated than that and speaks to another potential point of alliance: use of drugs and alcohol by the same individuals.

A variety of patterns of polysubstance use were identified in the workshops. Some participants described being omnivorous in their consumption of psychoactive substances. As one said, “I’m a crackhead, alcoholic, pothead; five months clean, but I smoke lots of pot to keep away from it. I’m trying to stay away from the booze, too, but I had some last night” (L). Others explained how alcohol had been a gateway drug to other substances: “It was in past years definitely opiates, but alcohol was the catalyst to all of it. I’d get totally run down on alcohol and flip flop back and forth for many years” (Q). Still others described a trajectory in the other direction; as one said, “I was addicted to cocaine… My drug of choice now is alcohol. When I do have money, that’s my downfall” (G).

Clearly, “illicit drinkers” and “drug users” as categories are neither exclusive nor stable. This reflects the created and fluid nature of the boundary between legal and (currently) illegal psychoactive substances. When combined with the common priorities and values described by participants, this polysubstance use provides further rationale for including illicit drinkers in drug user organizing.

## Discussion

### Barriers to collaboration between drug users and illicit drinkers

A unique contribution of this research was identifying the opportunity to involve illicit drinkers in drug users’ organizations based on shared priorities, shared values, and the realities of polysubstance use. Given the potential of this alliance, it is perhaps surprising that inclusion of illicit drinkers in drug user activism is not more widespread.^2^ We identified four potential reasons for this exclusion: racism, horizontal violence, extreme marginalization of illicit drinkers, and knowledge gaps around alcohol harm reduction.

#### Racism

In our needs assessment, illicit alcohol was most strongly raised as an issue for further exploration at workshops held in northern communities. This corresponds to provincial data showing that the highest per capita rates of alcohol consumption in British Columbia occur in the northern and interior regions and the northern part of Vancouver Island [22] and that alcohol abuse rates are higher among homeless people in the northern city of Prince George than they are in Victoria or Vancouver [23]. The proportion of attendees identifying as Indigenous also rose as we traveled north, until Indigenous attendees outnumbered non-Indigenous by a factor of two to one or more. Such a trend follows local demographics, as the northern regions of BC have proportionally more Indigenous residents.

In Canada and in many other colonial states, Indigenous peoples experience elevated proportions of substance use in general and alcoholism (and alcohol-related harms) in particular, although they also have higher proportions of non-drinkers [24–27]. The burden of substance use and mental health disorders in Indigenous communities is directly tied to the ongoing experience of colonialism, economic and political marginalization, and the legacy of residential schools [28, 29].

Indigenous people’s use of alcohol is framed differently in public discourses than non-Indigenous’. They are portrayed as having a genetic predisposition to the abuse of alcohol and in lacking control around its use and therefore in need of external controls to be placed on them [24]. These discourses serve the purposes of dominant groups by facilitating non-Indigenous control of Indigenous people and resources and the apprehension of Indigenous children [24, 30–32]. By creating the sense that Indigenous alcohol use is a “special case,” they may also hamper efforts to create links between drug users’ organizations and illicit drinkers.

One of the authors (LB) is the past president of the Western Aboriginal Harm Reduction Society (WAHRS), the world’s first Indigenous-specific harm reduction organization. WAHRS encourages participation from illicit drinkers, in contrast to the policy of most user-run harm reduction organizations, due to the large impact of alcohol on Indigenous communities and its intimate ties to histories of colonization, forced assimilation, and residential school systems. This suggests both that involvement of illicit drinkers in drug users’ organizations is possible, and that an acknowledgement of Indigenous-specific substance use issues may facilitate greater participation of illicit drinkers.

#### Horizontal violence

Horizontal violence is an idea whose origins lie in critical theory. It refers to oppressive acts committed by individuals or groups that are themselves marginalized and oppressed. When they are prevented from taking action against their oppressors, they may instead internalize the worldview of their oppressors and strike out against members of communities that are similarly lacking in power [33–35].

Horizontal violence has been described in the relationships between members of specific subgroups of drug users. Simmonds and Coomber [8] found that certain drug users characterize members of other subpopulations (for example, the non-homeless toward homeless or steroid users toward other drug users) as irresponsible in order to “displace acknowledgement” of their own risky behaviors and to minimize their own difference and stigmatization. Additionally, Radcliffe and Stevens [36] described how certain drug users receiving addiction treatment (such as women and cannabis users) used the pejorative label “junkie” to distance themselves from the stigma (and self-stigma) of using drug treatment services.

Horizontal violence is a potential factor that keeps drug users and illicit drinkers from working effectively together. Facilitators suggested that drug users can judge and even strike out at particularly marginalized people who drink alcohol when they are themselves experiencing discrimination and oppression. In addition, reflecting on their own perceptions of the inherent difficulty of working with drinkers, several facilitators concluded that many of their negative stereotypes of drinkers were based on a need to feel superior in their own choice of illicit substances. Challenging horizontal violence through consciousness-raising was seen, therefore, as a potential route to closer alliances in the future.

#### Extreme marginalization of illicit drinkers and the need for consciousness-raising

In Vancouver and in many other major cities around the world, leaders in the drug user communities and their allies have worked to politicize people who use illicit substances. This means awakening drug users to their own power to bring about lasting change and shifting focus from immediate needs (for example, daily food provision) to the institutions and political, economic, and social structures that influence how immediate needs are met [37–39]. It is necessary because the marginalization of illicit drug users creates a barrier to their engagement in self-advocacy. In the early days of the Vancouver Area Network of Drug Users, inspired by theories of popular education and liberation theology, community organizers encouraged drug users to recognize their power to effect change; as one founder put it, “The biggest obstacle to making the situation better was the marginalization of drug users, and the distance that addicts are from society. So the first thing we got involved in was the demarginalization of drug users” [37].

Illicit drinkers are an extremely marginalized population; a process of consciousness-raising may be necessary in order for them to take part in drug users’ organizations. Consciousness-raising is a term from the women’s movement that refers to a group process of sharing experiences and learning about how they are tied to systemic problems of power and oppression [40, 41]. A similar concept within critical theory is conscientization, “the process in which men [*sic*], not as recipients, but as knowing subjects, achieve a deepening awareness both of the socio-cultural reality which shapes their lives and of their capacity to transform that reality” [42]. The facilitator contrasted the situation of illicit drinker participants with those of Vancouver drug users now, who she felt had a consciousness of the links between the personal injustices they face and broader societal trends. This explained, according to her, the focus by illicit drinkers on small, immediate goals (such as longer shelter hours rather than expanded access to affordable supportive housing) and the lack of emphasis placed by drinkers on collective action as a strategy to achieve change. A process of engaging with illicit drinkers and developing their sense of their own political power could, then, promote full participation of illicit drinkers in user-run organizations.

#### Knowledge gaps

The majority of our workshop facilitators were experienced activists and leaders in the drug user community. As such, they felt very confident in their knowledge of harm reduction strategies for a variety of substances and routes of administration (injection, inhalation, etc.). This confidence did not, however, extend to their knowledge of harm reduction strategies for alcohol and particularly non-beverage alcohol. In particular, they felt that without a better understanding of the effects of non-beverage alcohol on the body, they could not advise drinkers on steps they could take to maintain their health. They believed this barrier could easily be overcome, however, through consultation with scientific experts and collaboration with experienced illicit drinkers.

## Conclusions

As part of a participatory assessment of drug users’ health and harm reduction needs, we identified three potential points of alliance between drug users and illicit drinkers: shared priorities, shared values, and polysubstance use. We also identified four potential barriers to collaboration: racism, horizontal violence, extreme marginalization of illicit drinkers, and lack of knowledge about alcohol harm reduction. Our results suggest that, although potentially challenging, involving illicit drinkers in drug users’ organizations has great potential for working toward the goals of both groups.

A limitation of this research was that participants were not a random sample of substance users. The networks used to recruit, particularly those of facilitators and social service organizations, influenced who attended. People who depend on social service agencies for survival often become entangled in organizational politics, and therefore previous experiences with an agency affect whether a person will attend an event with which they are affiliated. We did our best to make the workshops and meetings as low barrier as possible and therefore open to a broad range of participants (including those often excluded from research), however, by providing honoraria to support participation, not requiring sign-up or consent in advance, and holding meetings in locations accessible and familiar to substance users.

A strength of this research has been in its uptake and effects. Because it was conducted in partnership with an organization interested in acting on the results, we were able to quickly establish a follow-up research project focused on connecting with illicit drinkers in Vancouver’s Downtown Eastside, which subsequently led to the formation of an activist group for illicit drinkers, the Eastside Illicit Drinkers Group for Education (EIDGE). EIDGE’s ongoing work includes providing support and education to members through weekly meetings, partnering with a legal non-profit to create a “drinkers’ rights” card, participating in a national research project on managed alcohol programs, and advocating for a non-residential managed alcohol program in Vancouver.

Health care and harm reduction practitioners may incorporate this research into their practice by attending to opportunities to engage with people who drink non-beverage alcohol. In particular, it may be useful to expand harm reduction services for illicit drinkers and to explore how their specific needs in health care delivery can best be met. Drug users’ organizations should also consider how they may use their considerable expertise in activism and peer-based programming to support illicit drinkers in achieving their goals. Attention should be paid to the barriers that illicit drinkers face in becoming involved with self-advocacy for substance users and to minimize these barriers whenever possible. Illicit drinkers are some of the most marginalized members of society; expanded outreach to this group from health care providers and drug users’ organizations has the potential to contribute greatly to their improved well-being.

## Footnotes

1 The term “drug user” is used in this paper in addition to “person who uses illicit drugs.” This term is used to be in keeping with the terminology employed at the Vancouver Area Network of Drug Users, a partner organization for our research and to avoid the use of acronyms. We celebrate the fact that our participants are also family members, friends, workers, volunteers, and community members, in addition to drug users. Additionally, we call attention to Jauffret-Roustide’s use of the term, in contrast with the pejorative “drug addict,” as “a responsible, self-reliant citizen able to adopt preventive behaviors” [38].

2 There are undoubtedly exceptions to this generalization, of course. Of note, the Vancouver Area Network of Drug Users conducted a brief project in which people could exchange rice wine (non-beverage alcohol) for beverage alcohol [43].
